# Neuronal Nicotinic Acetylcholine Receptor Modulators from Cone Snails

**DOI:** 10.3390/md16060208

**Published:** 2018-06-13

**Authors:** Nikita Abraham, Richard J. Lewis

**Affiliations:** IMB Centre for Pain Research, Institute for Molecular Bioscience, The University of Queensland, St. Lucia, QLD 4072, Australia; n.abraham@imb.uq.edu.au

**Keywords:** conotoxins, α-conotoxins, nicotinic acetylcholine receptors

## Abstract

Marine cone snails are a large family of gastropods that have evolved highly potent venoms for predation and defense. The cone snail venom has exceptional molecular diversity in neuropharmacologically active compounds, targeting a range of receptors, ion channels, and transporters. These conotoxins have helped to dissect the structure and function of many of these therapeutically significant targets in the central and peripheral nervous systems, as well as unravelling the complex cellular mechanisms modulated by these receptors and ion channels. This review provides an overview of α-conotoxins targeting neuronal nicotinic acetylcholine receptors. The structure and activity of both classical and non-classical α-conotoxins are discussed, along with their contributions towards understanding nicotinic acetylcholine receptor (nAChR) structure and function.

## 1. Conotoxins—Venom Peptides from Marine Cone Snails

**Figure 1:**
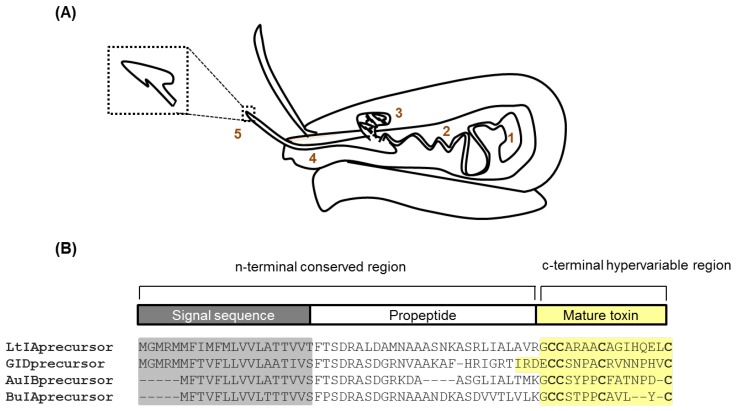
Conotoxin expression. (**A**) Conotoxins are produced in the venom apparatus that consists of (1) the venom bulb—a muscular organ that is used as a pump, (2) the venom duct which produces the venom, (3) the radular sac that stores the harpoons. Harpoons are evolutionarily modified radula teeth that are used to inject venom into the prey, like a hypodermic needle, (4) the proboscis is used to “load” (5) the harpoon at the tip to inject venom; and, (**B**) The venom peptides are translated as prepropeptide. They consist of a conserved N-terminal made up of the signal sequence and propeptide region which are cleaved during peptide processing. The final bioactive conotoxin consists of only the C-terminal region, which is also the disulfide rich hypervariable region of the prepropeptide.

Cone snails are marine gastropods belonging to the Conidae family, which are found in tropical, subtropical, and temperate waters around the world [1]. They use a specialized envenomation strategy to feed on worms (vermivorous), mollusks (molluscivorous), or fish (piscivorous) [2,3,4]. The venom apparatus consists of a duct expressing the venom components (Figure 1A), which are delivered intravenously via a hypodermic needle-like hollow, barbed harpoon that is held by the proboscis with the help of a muscular venom bulb at the other end of the duct [2,3]. The potency and rapid bioactivity of the venom is reflected by the small volumes injected (<~50 µL), which is sufficient for prey capture and defense, with some species being reported to cause human fatalities [1,5]. 

The cone snail venom is composed of a range of small molecules, peptides, and enzymes, however, it is the peptides that not only dominate the venom, but are also the neurotoxic components that are responsible for the rapid onset of the characteristic flaccid or spastic paralysis in the target [1,6,7]. Conopeptides have evolved to target a range of receptors, ion channels, and transporters in the central and peripheral nervous system [2,4,7]. In addition to this diversity in bioactivity, the venom compositions from the different cone snail species are also highly variable [2,8,9,10]. Initially, the estimated number of potential neuropharmacologically active compounds was around ~50,000 from 500 cone snail species [4,11]. Advances in highly sensitive transcriptomic and proteomic analyses of cone snail venom have shown that the peptides that are actively used by cone snails constitute less than 5% of the total number of peptides produced [1,12,13,14,15]. This arises through ‘variable peptide processing’, which produces single amino acid variants of a given peptide through deletions, frame shifts, stop codon shifts, variable N- or C-termini, and variable post-translational modifications to dramatically increase venom peptide diversity [1,12]. Evolutionarily, this strategy contributes to shifts in pharmacology through biological messiness to expand the pool of peptides with advantageous properties. Simultaneously, it has also made available a natural combinatorial library providing a rich natural source of peptides that is useful to dissect properties of mammalian membrane proteins, especially ion channels.

There are two broad groups of *Conus* venom peptides: the disulfide poor and disulfide rich peptides [16]. The disulfide poor peptides include the contulakins (targeting the neurotensin receceptor), the conantokins (targeting the *N*-methyl-d-aspartic acid receptor), the conoformides (targeting the Rfamide receptor), the conophans (target unknown), the conomarphins (target unknown), the contryphans (target unknown), and the conopressins (vasopressin homologs) [2,16]. The disulfide rich peptides are the conotoxins, which are a structurally and functionally diverse class of peptides that target ion channels with high potency and selectivity [2,16].

Conotoxins are translated as prepropeptides consisting of a highly conserved N-terminal signal sequence, followed by a conserved pro region that separates the signal sequence from the highly variable, disulfide rich, C-terminal mature toxin sequence [16]. The prepropeptide undergoes proteolytic cleavage to yield the mature bioactive toxin, which is generally 12–30 amino acid residues in length (Figure 1B). Conotoxins are classified into superfamilies, which include peptides that share the same conserved signal sequence. These are further divided into families, which include peptides with a similar cysteine framework and homologous pharmacological targets [2,4,13]. The high degree of variations in potency and selectivity between peptides within families arises through single amino acid changes in regions between the conserved cysteine residues.

**Figure 2:**
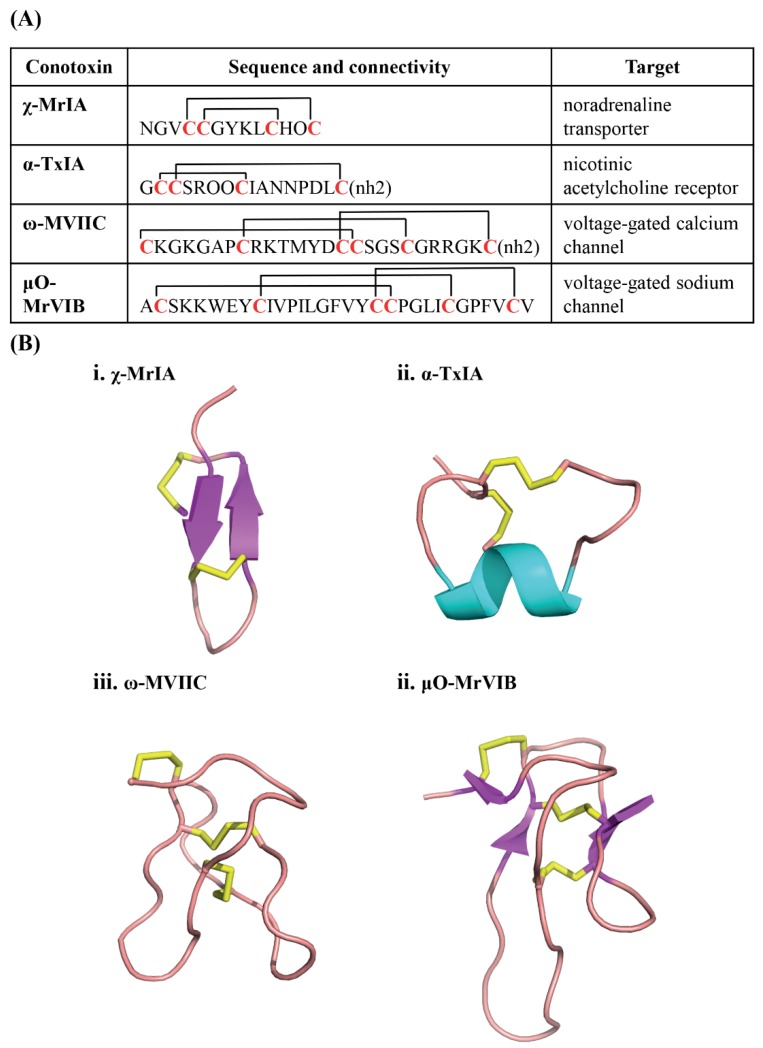
Conotoxin structural and functional diversity. (**A**) Conotoxins have highly diverse sequences, cysteine frameworks, connectivity, and biological targets. χ-MrIA and α-TxIA have the same cysteine framework, but different connectivities and sequence which contribute to the difference in their targets. On the contrary, ω-MVIIC and μO-MrVIB have the same cysteine framework and connectivity but different biological targets. (O = hydroxyproline); (**B**) Conotoxin diversity extends to their three-dimensional structures, and thereby further adding to their functional diversity.

The three-dimensional structure of conotoxins in general have a rigid backbone due to the restraining cysteine residues that help to stabilise well-defined secondary structural elements like α-helices, β-sheets and turns. As expected, the three-dimensional structure has an important influence on conotoxin bioactivity. For example, conotoxins χ-MrIA belonging to the T superfamily and α-TxIA belonging to the A superfamily have similar number of cysteine residues (cysteine framework), but different disulfide connectivity. This difference in connectivity underlies their different three-dimensional structures [17,18,19], where χ-MrIA targets the noradrenaline transporter and α-TxIA targets the nicotinic acetylcholine receptors (nAChRs) (Figure 2) [20,21]. On the contrary, ω-MVIIC and μO-MrVIB are examples of peptides that have same disulfide connectivity, but have very distinct structures and pharmacological targets (Figure 2) [22,23]. The diversity in three-dimensional structures underpins the usefulness of conotoxins as highly selective molecular probes and templates for rational drug design.

Conotoxins belonging to the α, ω, µ, µO, δ, σ, κ, χ families are widely expressed across cone snail species, and have been pursued for their clinical potential in pain management, epilepsy, cardiac reperfusion [2]. This review will focus on the pharmacology, structure-function and applications of the α-conotoxins.

## 2. α-Conotoxins

**Figure 3:**
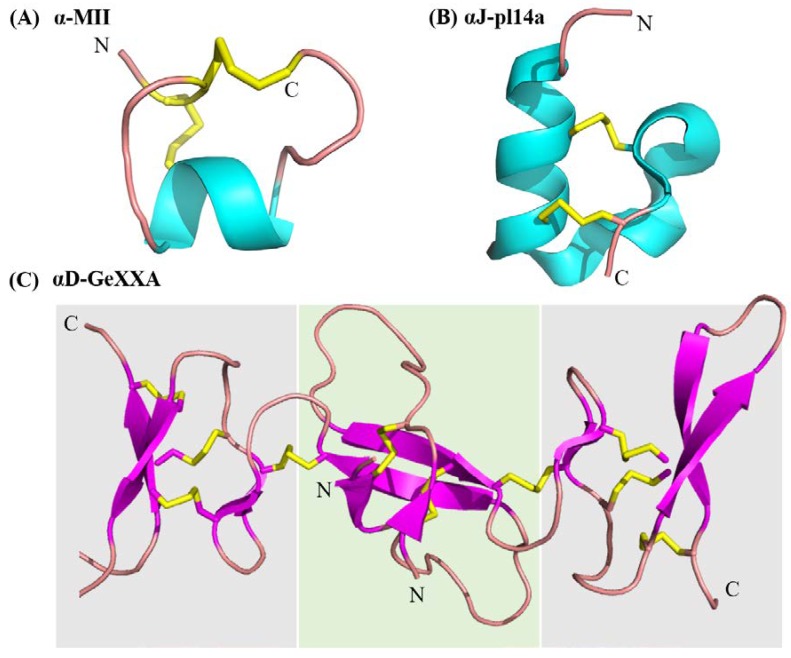
Structural diversity in conotoxins modulating the nicotinic acetylcholine receptors (nAChRs). (**A**) α-Conotoxin MII representing a ‘typical’ globular α-conotoxin structure with a CC−C−C framework, I–III II–IV connectivity and α-helical backbone (**B**) αJ-pl14a—a non-classical nAChR modulator from the J superfamily, with a C−C−C−C framework, I–III II–IV connectivity resulting in a very different structure and potentially binding mode and nAChR interactions. (**C**) αD-GeXXA a representative of the D superfamily, which are natively dimeric modulators of nAChRs. The C-terminal domain of each monomer is shown against a grey background and the N-terminal is against a green background. Contrary to the α-helical motifs seen in other nAChR specific conotoxins, αD-GeXXA primarily consists of β-sheets. The unusual structure is also associated with a very different receptor modulation mechanism. This figure represents only a fraction of the diversity associated with conotoxin modulators of nAChRs. Table 1 provides further sequence and functional details for conotoxin modulators of nAChRs, whose structures have not been determined.

Conotoxins specifically targeting the nicotinic acetylcholine receptor (nAChR) mostly belong to the A superfamily and are denoted with an ‘α’ to indicate nAChR specific activity [24,25,26,27]. However, nAChR specific conotoxins have also been isolated from the B3, D, L, M, O1, S, T, and J superfamilies as well, and are denoted by an ‘α’, followed by the superfamily e.g., αD-VxXXB [26,27,28,29,30,31,32,33]. α-Conotoxins from the various superfamilies have distinct cysteine frameworks (Figure 3) and three-dimensional structures, and they are the largest group of conotoxins characterised [34], highlighting the importance of nAChRs as targets [3]. nAChRs play important roles in neuronal signaling, especially the α1β1γδ/ε and α7 nAChR subtypes that are major modulators of neurotransmission across the neuromuscular junction and in the central nervous system, respectively [35,36]. The α7 subtype is also capable of inducing downstream signaling mechanisms in non-neuronal cells, and is thought to be an ancestral form evolved in lower organisms that do not rely on fast excitatory mechanisms [37,38,39,40,41]. Consequently, it is hardly a surprise that α-conotoxins are so abundantly expressed in cone snail venom, majority of which target the muscle α1β1γδ/ε nAChR or the neuronal subtypes, including the α7. The unique structural features of α-conotoxins allows for them to differentiate between the muscle and neuronal nAChR subtypes—a property that distinguishes them from other classes of natural product inhibitors of nAChRs that predominantly target the muscle subtype or exhibit poor selectivity between muscle and neuronal subtypes [2,24,42,43].

Classical α-conotoxins are 12–20 amino acids long with four cysteine residues that are arranged in a CC−C−C framework, which can result in the globular (C1−C3 and C2−C4) ribbon (C1−C4 and C2−C3) and bead (C1−C2 and C3−C4) disulfide isomers [11,42,43,44]. The globular isomer is the naturally occuring bioactive form, while the ribbon and bead are typically either weaker inhibitors or inactive [18,21]. Based on the number of amino acids between these cysteine residues, α-conotoxins are further classified as the 3/5, 4/3, 4/4, and 4/6 types [2,11,45]. Of these, the 3/5 α-conotoxin sub-group specifically block the muscle nAChRs. The 4/3, 4/4, 4/6 4/7, and most recently, 5/5 (AusIA) are neuronal nAChR antagonists with no obvious correlation between the sub-group and nAChR subtype specificity [11,45]. α-Conotoxins also undergo post-translational modifications, with the C-terminal amidation occurring commonly. Loss of the C-terminal amidation has been shown to disrupt the three-dimensional structure leading to a decrease in bioactivity, a phenomena that are consistently observed in peptides that were isolated from venomous animals, such as spiders, scorpions, wasps, and others [18,46]. Structurally, the α-conotoxins are rigid molecules due to the restraining disulfide bonds, conserved proline residue in loop 1 and a short 3_10_ α-helical backbone (Figure 3) [45].

## 3. α-Conotoxin Contributions to Determining nAChR Ligand Recognition Properties

The nAChRs are ligand gated ion channels falling under into two broad categories (1) the muscle nAChR subtype (α1β1γδ/ε) that modulates neurotransmission at the neuromuscular junction and (2) the neuronal nAChR subtypes, which are significantly more diverse than the former, which modulate neurotransmission in the central and peripheral nervous systems [47,48]. This review focuses on α-conotoxins specifically inhibiting the neuronal nAChR subtypes. Neuronal nAChRs, such as the α7, α9α10, α4β2, α3β2, α3β4, and α6 containing subtypes are therapeutic targets for Alzheimer’s, Parkinson’s, drug addiction, lung, and breast cancers [47,48,49,50,51]. The therapeutic potential of nAChRs lies in the ability to selectively target the subtype that is associated in the given condition. However, this is challenged by the high degree of homology between the nAChRs in sequence, structure, and ligand recognition properties [52,53,54,55,56]. The selectivity window of a ligand for a given nAChR subtype is greatly influenced by subtle variations in the pair-wise receptor-ligand interactions [57,58]. Advances in our understanding of the variations in ligand recognition properties of the different nAChR subtypes, receptor biochemistry and pharmacology has been greatly facilitated by α-conotoxins. This is due to a combination of factors, such as (1) a large (>50) combinatorial library of peptide sequences, (2) the inherent potency and specificity of α-conotoxins, (3) the broad range of subtypes targeted with varying selectivities, (4) the relative ease of synthesis that form the foundation for extensive structure-activity studies, and (5) ability to obtain high resolution structures of the conotoxins alone and in complex with the nAChR homologue—the acetylcholine binding protein (AChBP) [59]. AChBP is a soluble protein, which modulates neurotransmission in molluscs and worms, by sequestering acetylcholine released in the synapse [60,61]. AChBP and the nAChR extracellular, ligand-binding domain have remarkable homology, exhibiting conserved features, such as the β-sandwich core, the cys-loop, and the vicinal disulfide on the C loop capping the ligand binding pocket [62]. Most importantly, they both share the conserved aromatic cage residues responsible for orthosteric ligand recognition in nAChRs and both bind nAChR ligands, including the α-conotoxins similarly [62]. Recent high-resolution structures of the neuronal nAChR have validated the continued use of AChBPs as a high-througput, relatively less labor intensive approach of structurally investigating receptor-ligand interactions [56,63,64,65]. 

**Figure 4:**
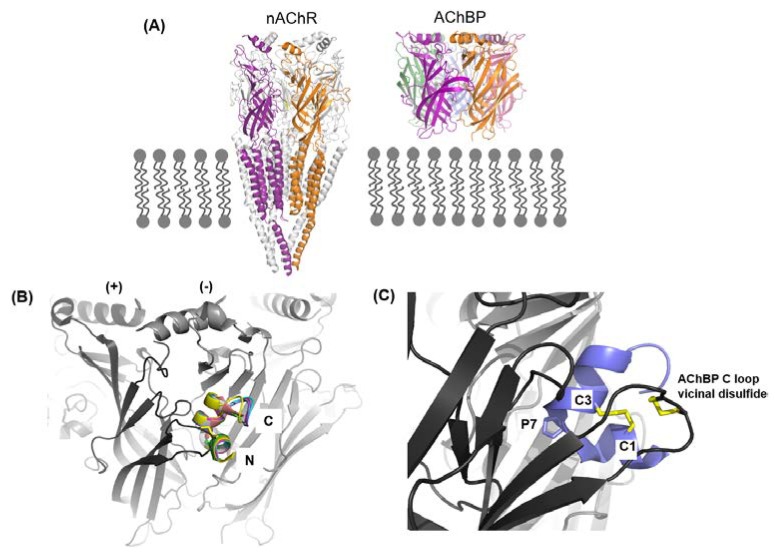
α-Conotoxin binding mode. (**A**) The acetylcholine binding protein (AChBP) is a soluble homologue of the nAChR ligand binding extracellular domain. AChBP is used extensively as an nAChR surrogate in receptor-ligand structure-activity studies; (**B**) An overlay of AChBP and ImI, PnIA (A10L, D14K), TxIA, LsIA, BuIA, GIC, PeIA, and LvIA co-crystal structure show a common binding mode at the interface of the principle (+) and complementary (−) subunits; (**C**) The conserved α-conotoxin residues responsible for anchoring the α-conotoxins in the binding pocket are shown.

Co-crystal structures of AChBP with α-ImI (PDB code 2BYP), α-PnIA variant (2BR8), α-TxIA (2UZ6), α-LsIA (5T90), α-BuIA (4EZI), α-GIC (5CO5), α-PeIA (5JME), and α-LvIA (5XLG) [66] demonstrate that α-conotoxins bind at the orthosteric binding pocket with the N-terminal oriented to the membrane side and C-terminal oriented to the top of the channel and the α-helical backbone buried within the aromatic cage (Figure 4B,C) [67,68,69,70]. The α-conotoxins are anchored into the binding pocket by the conserved α-conotoxin proline in loop 1, wedged deep within the aromatic pocket (Figure 4C). The 1–3 disulfide bond always stacks onto the vicinal disulfide of the C-loop, and it is perhaps the reason for the bioactivity of the globular α-conotoxin isomer over the ribbon and bead forms [67,68,69,70]. Unlike the small molecule nAChR ligands, which only interact with the conserved nAChR ligand binding residues, α-conotoxins additionally interact with the variable residues outside this conserved aromatic core [57,58,67]. 

Despite α-conotoxins sharing a common binding mode that is underpinned by a few conserved interactions, the majority of the pair-wise receptor-ligand interactions are highly variable [67,71]. This variability is the driving force underlying the different pharmacological profiles of α-conotoxins. For example, PnIA, PnIB, ImI, LsIA, and MrIC are all active at the α7 subtype [72,73,74,75,76]. However, each has a different potency and contrary to the activity of others, MrIC acts as a state-dependent agonist of the α7 [77,78]. This is despite the high likelihood of MrIC also adopting the typical α-conotoxin binding mode at the orhtosteric binding pocket, as demonstrated by its typical α-conotoxin structure and its ability to displace radiolabeled orthosteric ligands [77]. PnIA and PnIB differ by only two amino acid residues, which is sufficient to alter the selectivity profile. PnIB is more selective for α7 over the α3β2, whereas PnIA is a relatively more selective α3β2 inhibitor [72,73,74]. Similarly, LsIA inhibits the α7 with 10 nM potency, however, the substitution of just two residues alters its interaction with the (−) face of the receptor binding pocket, such that it loses complete activity at the α7, and instead selectively and potently inhibits the α3β4 subtype [69,76]. Another example can be seen in α-conotoxin BuIA, which is a rare 4/4 α-conotoxin and one of the very few α-conotoxins that is selective for the α6* subtype. Despite its unique features, the co-crystal structure (4EZI) shows BuIA adopting the typical α-conotoxin binding mode. Thus, reinforcing the significance of the pair-wise interactions between α-conotoxin and nAChR forming the foundation for its subtype selectivity. 

**Figure 5:**
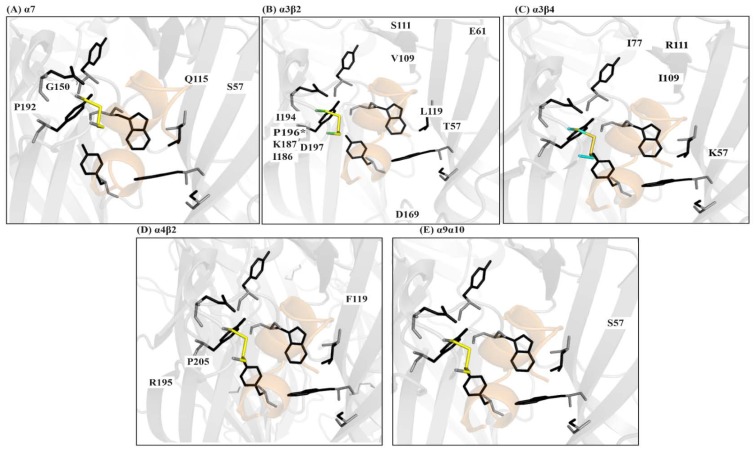
nAChR selectivity determinants identified using α-conotoxins. The α-conotoxin backbone wedges deep within the conserved aromatic core in the nAChR binding pocket. The side chains extend beyond this core and engage variable residues on the (+) and (−) face of the binding pocket to obtain subtype-selectivity. A representative α-conotoxin is shown surrounded by the residues forming the aromatic core as sticks (black). Subtype selectivity determinants identified using α-conotoxins and their locations outside the core are represented by residue labels. (**A**) Residues modulating α7 selectivity [93,94,95,96]; (**B**) Residues modulating α3β2 selectivity [70,79,82,97]. P196 modulates species selectivity for the α3β2 [82]; (**C**) The α3β4 selectivity is largely modulated by residues in the (−) face of the binding pocket [69,84,85]; (**D**) Residues modulating α4β2 selectivity [79,92]; and, (**E**) Interactions with S57 is critical to obtain α9α10 species selectivity. α-Conotoxin interactions with this residue were key in determining the α9α10 stoichiometry as α10(+)α9(−), contrary to the previously assumed α9(+)α10(−) [98].

The significance of these pair-wise nAChR-conotoxin interactions are now being understood through co-crystal structures, homology modelling and docking, site directed mutagenesis, and alanine scans combined with pharmacological evaluations of these modifications to define the potency and selectivity determinants of the different nAChR subtypes. For example, Dutertre et al. used a computational approach to demonstrate the interactions of α-conotoxins MII, PnIA, and GID with a binding cleft on the β2 subunit of the α3β2 subtype as the basis for their pharmacological profile [79]. Similarly, a co-crystal structure α-TxIA and AChBP was used to identify a distinct binding orientation, resulting in a key interaction between the amino acid at position 5 and the α3 subunit that is responsible for TxIA’s selectivity for α3β2. Pharmacological characterisation of native peptide LvIA identified another putative interaction between position 11 and the α3 subunit (assuming the typical α-conotoxin binding mode) as the basis for its α3β2 selectivity [80,81]. Taken together, these studies identify the key residues and interactions modulating α3β2 selectivity. Kompella et al. used systematic mutagenesis of RegIIA to demonstrate that pair-wise receptor-ligand interactions can also influence selectivity between species, identifying a single amino acid variation between the rat and human α3β2 subtypes rendered RegIIA, and its analogue 70-fold less potent at the human subtype (Figure 5B) [82].

Similarly, α-conotoxin TxID was characterized as the most potent inhibitor of the α3β4 subtype and position 9 on the peptide was reported to be critical for its activity [83,84]. Our structural investigations revealed that position 9 interacts with charged residues on the β4 subunit. We further demonstrated that interactions, such as cation-π, between an aromatic group on the peptide and charged residues on the β4 subunit are favourable for potent inhibition of this subtype [69]. Similarly, alanine scan mutagenesis of RegIIA identified a relatively conserved –NN– motif to be important for its activity at the α3β4 [85]. Our work demonstrates that this motif primarily modulates α3β4 activity through the first asparagine residue of the motif and its interactions with the β4 subunit [69]. Together, these studies show that engaging residues on the β4 subunit is key to obtaining selective inhibition of the α3β4 subtype (Figure 5C). 

Similar studies using α-conotoxins selective for some of the major nAChR subtypes, such as the α9α10 [43,86,87], α4β2 [79,88,89,90,91,92], and others have been instrumental in providing a blueprint of the molecular factors that are required for the potent and selective inhibition of these subtypes [2,24,42,43,71] (Figure 5A–E). Together, they provide the information on minimum pharmacophores that are required for the rational development of selective therapeutics and molecular probes.

## 4. The Non-Classical α-Conotoxins

As discussed above, classical α-conotoxins have provided major insight into orthosteric ligand recognition at the nAChRs and pair-wise interactions defining subtype selectivity. In contrast, non-classical α-conotoxins with unusual pharmacology, binding site interactions, cysteine connectivity, and three-dimensional structures can help to gain insight into novel facets of nAChR structure and function, including its allosteric regulation (Table 1).

### 4.1. α-Conotoxins from the A Superfamily Exhibiting Unusual Characteristics

AuIB: AuIB is a 4/6 α-conotoxin identified from *C. aulicus*, with 15 amino acids and the typical cysteine framework. Interestingly, the native globular AuIB is a non-competitive inhibitor of the α3β4 subtype [99]. Molecular docking studies identified two potential binding pockets above and below the orthosteric binding pocket, although these remain to be validated [100]. Contrary to typical α-conotoxin behavior, the AuIB ribbon isomer is 10-fold more potent in rat parasympathetic ganglia than the globular isomer, despite possessing a less defined three-dimensional structure when compared with the globular isomer [99]. Moreover, unlike the non-competitive globular isomer, the ribbon isomer is a competitive inhibitor of the receptor showing subunit stoichiometry dependence [100]. 

AusIA: AusIA is a recently discovered α-conotoxin from the venom of *C. australis*, defining a new 5/5 subclass [101]. Interestingly, both the globular and the ribbon isomers were found to be bioactive, with the ribbon isomer inhibiting the α7 subtype ~2-fold more potently than the globular isomer [101], demonstrating that the disulfide pattern is not as critical to the activity of this peptide as for the classical α-conotoxins.

ImII: α-Conotoxins ImI and ImII were both identified from the venom of *C. imperialis* [75]. Both peptide share 75% sequence similarity, differing by only three out of the 11 residues [102]. The three-dimensional structures of the two peptides are also highly similar and both inhibit the α7 subtype equipotently. Interestingly, while ImI inhibits the receptor via the orthosteric binding pockets, ImII has been shown to target a different binding pocket [102]. It is noteworthy that the conserved proline residue in loop 1, which is responsible for anchoring α-conotoxins in the orthosteric binding pocket, is substituted by an arginine in ImII that perhaps contributes to its allosteric mode of action [102].

LtIA and Lp1.1: α-Conotoxins have a highly conserved Ser-Xaa-Pro in loop 1. α-Conotoxins without this motif are inhibitors of the muscle nAChR with the exception of LtIA and Lp1.1 [103,104]. LtIA isolated from *C. literratus* and Lp1.1 isolated from *C. leoperdus* have a Ala-Xaa-Ala motif and inhibit the neuronal α3β2 and α6α3β2β3, and both are characterized by a fast off rate relative to the Ser-Xaa-Pro containing MII [103]. Molecular modelling and docking studies that were performed on LtIA suggest that while it is a competitive blocker, the binding site is relatively shallower when compared to that of a typical α-conotoxin [103]. This conserved motif is also absent from α-RegIA/f detected in *C. regius* using transcriptomic approaches, although it remains to be seen whether the α-RegIA/f pharmacological and structural properties are consistent with those that were seen for LtIA and Lp1.1 [105]. 

MrIC: MrIC was identified from *C. marmoreus* using transcriptomic approaches, and has the typical α-conotoxin cysteine framework and three-dimensional structure [77]. Interestingly, MrIC was shown to be an agonist at the α7 nAChR in the presence of type II nAChR positive allosteric modulator, suggesting that the MrIC had unique receptor state dependence [77,106]. Given that the cysteine framework, disulfide pattern, three-dimensional structure, and potentially the binding mode conform to that of typical α-conotoxins, this unusual behavior is likely to be driven by the primary sequence differences. Indeed, along with sequence variations in loop 1 and 2, MrIC has an extended hydrophobic N-terminal. However, the precise molecular factors contributing to this unusual pharmacology remain to be identified. 

Eu1.6: Liu et al. recently reported the discovery and characterization of Eu1.6 from *C. eburneus*, an α-conotoxin inhibiting Cav 2.2 [107]. Eu1.6 is 16 amino acids long with the characteristic α-conotoxin cysteine framework and typical globular isomer three-dimensional structure. Despite the hallmark α-conotoxin features, Eu1.6 has been reported to only weakly inhibit the α7 and α3β4 nAChRs, and instead is a potent inhibitor of Cav2.2 exhibiting analgesic activity in rat partial sciatic nerve injury and chronic constriction injury pain models [107].

### 4.2. α-Conotoxins from Other Superfamilies Targeting nAChRs 

nAChR specific conotoxins classified under the B3, D, L, M, O1, S, T, and J superfamilies show highly diverse cysteine frameworks, mode of action and three-dimensional structures (Figure 3 and Table 1). These unconventional α-conotoxins inhibiting the neuronal subtypes are discussed further below. 

#### 4.2.1. S Superfamily

Two nAChR inhibitors have been identified from this superfamily, namely αS-RVIIIA from *C. radiatus* and αS-GVIIIB from *C. geographus* [9,30]. RVIIIA was the first nAChR inhibitor to be isolated from a non-A superfamily. It is a 47 amino acid long peptide with 10 cysteine resides, although the disulfide connectivity is unknown [9]. While the common C-terminal amidation is absent, RVIIIA is post-translationally modified showing the presence of two γ-carboxyglutamates. RVIIIA is a competitive inhibitor with a broad selectivity that irreversibly inhibits the muscle subtype, but reversibly inhibits neuronal α7, α6/α3β2β3, α3β2 subtypes, and to a lesser extent α3β4 and α4β2 [9]. In contrast, αS-GVIIIB almost exclusively inhibits the α9α10 subtype [30]. αS-GVIIIB also contains 10 cysteine residues (disulfide connectivity unknown), although it is shorter relative to RVIIIA and has an amidated C-terminus [30]. αS-GVIIIB was reported to be a competitive inhibitor, binding at the orthosteric binding site at the interface of the α10/α9 subunits, although the specific binding mode and interactions are yet to be determined. 

#### 4.2.2. D Superfamily

VxXXA, -B, and -C were the first D superfamily peptides that were identified, and were first isolated from the venom of *C. vexillum* [27]. Similar to the S superfamily conotoxins, VxXXA, -B and -C comprise 47 amino acids with ten cysteine residues and post-translational modifications, including hydroxyproline and γ-carboxyglutamic acid. Interestingly, αd-conotoxins natively exist as 11 kDa covalently linked homo-dimeric proteins. VxXXA, -B, and -C inhibit the α7, α3β2, and α4β2, with VxXXB being the post potent, inhibiting the nAChRs with low nanomolar affinity (α7 0.4 nM, α3β2 8.4 nM, α4β2 228 nM) [27]. Furthermore, radioligand binding studies on AChBP revealed that these αd-conotoxins were allosteric inhibitors of the nAChRs [27]. cDNA evidence revealed that αd-conotoxins were encoded in a number of related species, including *C. capitaneus*, *C. mustelinus*, and *C. miles* [32]. Recently, αD-GeXXA from *C. generalis* was expressed and characterized. Like the αDs from *C. vexillum*, αD-GeXXA was also dimeric with each monomer consisting of ten cysteine residues [33]. However, αD-GeXXA exclusively inhibited the α9α10 subtype and were not potent inhibitors of α7 and α3β2, indicating that sequence variation in the large αd-conotoxins again contributes to unique pharmacological profiles. 

The high-resolution crystal structure of αD-GeXXA has provided unique insights into this dimeric class of conotoxins [33]. Each monomer was found to have an N- and C-terminal domain. The N-terminal region comprised loops and beta-sheets, and the disulfide bonds responsible for dimerization. This N-terminal core was flanked by the C-terminal domains, which although lacking specific secondary structure, was compact and rigid due to three restraining disulfide bonds adopting the canonical inhibitory cysteine knot [33]. The N- and C-terminii are also held stable relative to each other via a disulfide bond. Interestingly, when synthesized as a monomers, the N- and C- termini retained inhibitory activity independent of each other, albeit with reduced potency when compared to the native dimeric form. Based on receptor mutagenesis and systematically truncated analogues, the αD-GeXXA binding mechanism is proposed to involve the two C-termini of the dimer spanning across the top of the extracellular domain of the receptor to interact with residues on the α10 subunits, which results in the N-terminii ‘covering’ the pore of the nAChR [106]. This is proposed to stabilize the inactive/resting state of the receptor, preventing the global conformational changes that are required for channel activation [106], thereby explaining its allosteric pharmacology. Given the αDs from different species have different nAChR subtype selectivity, identifying the pharmacophores for other αDs could provide novel, allosteric strategies of subtype selective nAChR inhibition. 

#### 4.2.3. B3 Superfamily

α-VxXXIVA is the founding member of the B3 superfamily with a novel cysteine framework C−CC−C [26]. Identified using the cDNA library that was prepared from *Conus* venom ducts, this peptide is forty amino acids long and was found to inhibit the α9α10 with low micromolar affinity. The potency of α-VxXXIVA was found to be dependant on the disulfide connectivity, with 1.2 µM > [1,3] 3.9 µM > [1,4] >30 µM, suggesting that the novel cysteine framework is important for its mode of action [26]. However, the peptide mostly lacked an ordered three-dimensional structure, as seen from 1D H^1^ NMR and CD spectroscopy, except for the VxXXIVA [1,2] isomer, which showed about ~50% α-helical and β-sheet secondary structure in the presence of 87% TFE [26]. Although the low affinity and the lack of ordered structure are a challenge to determine a potentially novel mode of nAChR inhibition by α-VxXXIVA, it is a good example of how disulfide content and framework can influence the pharmacological profile and present novel opportunities for rational chemical synthesis of new inhibitors. 

#### 4.2.4. O1 Superfamily

Conotoxins belonging to the O superfamily are well known antagonists of the voltage gated calcium channels. While the precursor signal sequence of GeXIVA is similar to the O1 superfamily, it is a potent inhibitor of the α9α10 nAChR subtype with no activity at the Ca_v_ channels up to 1 µM [29]. Contrary to the 6-Cys pattern that was found in conotoxins from this superfamily, the GeXIVA mature peptide has only four cysteine residues. The bead isomer is most potent (4.6 nM), followed by the ribbon isomer, with the generally favoured globular isomer being least potent—a trend that is opposite to that of classical α-conotoxins. The ribbon and bead isomers also had a more ordered three-dimensional structure when compared to the globular isomer. GeXIVA is highly charged, with nine arginine residues among the total 28 residues making up the peptide, and suggested to be an allosteric inhibitor based on voltage-dependent activity and kinetics of disassociation [29]. 

#### 4.2.5. T Superfamily

Peptides belonging to the T superfamily have CC−CC cysteine framework and mainly target the voltage gated ion channels [2]. Wang et al. recently reported the discovery and characterization of a novel conotoxin TxVC, which has the T superfamily cysteine framework and inhibits the α4β2 and α3β2 nAChRs, with no activity at the voltage gated ion channels [31]. Since gene or mRNA data were not identified, TxVC cannot be confirmed to belong to the T superfamily based on cysteine framework alone [108]. Nevertheless, TxVC presents a novel primary sequence and disulfide connectivity and one of the very few nAChR specific conotoxins with activity at the α4β2, making it a useful tool to dissect the property of this nAChR subtype that is expressed in the brain. Based on site-directed mutagenesis, the pharmacophore of TxVC appears to overlap that of the α4β2 specific α-conotoxins GIC and GID [31]. However, the variation in primary sequence, disulfide connectivity and three-dimensional structure from typical α-conotoxins suggest that TxVC likely adopts a different binding pose.

#### 4.2.6. J Superfamily

Unlike the conotoxin discussed so far, which primarily target the nAChRs, pl14a was the first conotoxin that is characterized to inhibit both the voltage and ligand gated ion channel. Isolated from *C. planorbis*, pl14a defined a new superfamily J, with a C−C−C−C framework and 1–3, 2–4 connectivity. pl14a inhibits Kv1.6 (IC_50_ = 1.59 μM), as well as the muscle α1β1εδ (IC_50_ = 0.54 μM) and neuronal α3β4 (IC_50_ = 8.7 μM) nAChR [28]. Its three-dimensional structure includes a 3_10_ α-helical backbone and a flexible extended N-terminus. Structural similarities between the backbone conformation for residues 11–21 in pl14a and the conformation observed for residues 4–12 of classical α-conotoxin were observed, suggesting that this region might be relevant for its activity at the nAChRs [28].

#### 4.2.7. M Superfamily

Peptides belonging to the M superfamily have a CC−C−C−CC cysteine framework. Like J superfamily conotoxins, µCnIIC from *C. consors* inhibits both voltage-gated (Nav 1.2 and 1.4) and ligand-gated (α3β2 nAChRs) ion channels [109], albeit with weaker activity at nAChR. Presently, it is not clear whether µCnIIIC inhibits nAChRs orthosterically or allosterically. A high resolution structure of this peptide is reqired to help understand what structural features could potentially explain its ability to target two classes of receptors [109]. 

In conclusion, as evidenced by the remarkable diversity in the structure, activity, and binding modes of the non-classical α-conotoxins, it is clear that cone snails have developed multiple strategies to interfere with the global conformational changes that are involved in activating the nAChRs. However, the size, complexity, and unknown disulfide connectivity of most non-classical α-conotoxins makes them challenging for chemical synthesis. Nevertheless, breakthroughs are being made in this aspect, as evidenced by the work of Yang et al. (2017), increasing the potential of these α-conotoxins to contribute to the discovery of new ligand recognitions mechanisms at the nAChRs [106].

## 5. Applications

As discussed above, the leading application of α-conotoxins continues to be for structure-function studies to identify both orthosteric and allosteric mechanisms of nAChR ligand recognition and modulation. However, α-conotoxin applications also extend beyond this primary role, including the selected applications outlined below. 

(1) Molecular probes

α-Conotoxins are the largest group of natural product peptide inhibitors of the nAChRs [2,7,34]. While the majority of natural product inhibitors of nAChRs target major α1β1γδ/ε and α7, α-conotoxins have broader selectivity even targetting some of the most elusive neuronal nAChR subtypes, such as α6α3β2β3, α3β4, and α9α10 [2,108,110,111]. This makes both the native and the rationally optimised α-conotoxin analogues excellent tools to elucidate the physiological and pathological functions of these subtypes. For example, α-conotoxin MII identified α6 containing nAChR subtypes (α6α3β2β3) as regulators of dopamine release [45,112,113]. Since dopaminergic mechanisms modulate learning, psychotic and addictive behaviour, motor co-ordination, and Tourette’s syndrome [45,114], the identification of α6α3β2β3 nAChRs as major modulators of dopamine release provides new insight into the pathophysiology of these disorders. Similarly, a recent study utilized α-conotoxin AuIB—one of few antagonists selective for the α3β4 nAChR, to demonstrate that the selective block of this subtype can be an efficient therapeutic strategy to prevent the progress of lung cancer [115]. While α-conotoxins are largely synthesised in-house using established methods, rationally improved, selective analogues of AuIB, ImI, PIA, PeIA, Vc1.1, and BuIA are commercially available as research tools.

(2) Identifying species variation in nAChRs

Although nAChR subtypes are notoriously homologous, important sequence variations occur that can influence ligand potency and selectivity for a particular subtype from different species. This can have important implications in drug design and development, as well as the development of agrochemicals, insecticides, and pesticides. α-Conotoxin Vc1.1 entered a human clinical trial for chronic pain, however, the trail was halted after phase-2A over concerns of lower potency at the human α9α10 nAChR [98]. Similarly, RgIA was found to exhibit >300-fold lower potency at the human α9α10 versus rat subtype [98]. Subsequently, a single residue substitution of threonine at position 56 (rat) to an isoleucine (human) was found to be responsible for this species variation at the α9α10 nAChR [98]. Presently, the majority of the α-conotoxin pharmacology has been performed on mouse/rat receptors and extrapolations to the human receptors need to be done with caution [2]. A broader structural and pharmacological evaluation of species variation exhibited by α-conotoxins could provide valuable insights and can have important implication in the development of therapeutics and agrochemicals. 

(3) α-Conotoxin re-engineering

The relative ease of α-conotoxin synthesis has enabled the exploration of novel strategies to improve the pharmacological properties of peptide ligands using the α-conotoxin scaffold as a template. Such approaches have led to the development of selenocysteine derivatives, cyclised variants, and lipoamino acid 2-amino-d-l-dodecanoic acid coupled analogues [116,117,118,119,120]. These modified α-conotoxin variants are more resistant to reduction, disulfide scrambling, enzymatic degradation, and have improved oral bioavailability, properties that generally put peptides at a disadvantage of being developed as therapeutics [121,122]. The cyclised α-conotoxin scaffold was used to engineer glucagon-like peptide-1 peptidomimetics that are targeting the glucagon-like peptide-1 receptor that is implicated in type-2 diabetes mellitus [123]. The resulting molecule was reported to have increased hydrophobicity that can improve membrane permeability and bioavailability [123]. Indeed, multimeric α-conotoxins have been developed to enhance α-conotoxin potency and selectivity at homomeric nAChRs [124]. Alkyne-polylysine dimers of ImI, an antagonist of α7 and α3β2 nAChRs [102], had enhanced activity at the homomeric α7 when compared with the heteromeric α3β2 subtype, providing a novel strategy to improve upon the selectivity and potency properties of α-conotoxins. Peptide dendrimers are of emerging interest with the potential for slowed renal clearance due to their molecular size, in addition to enhanced activity due to their multivalency effect [124]. 

(4) Targeted drug delivery

A recent study exploited the inherent high potency and specificity of α-conotoxins for nAChRs in order to obtain targeted drug delivery. Targeting α7 nAChRs over-expressed in breast cancer, the α7 selective ImI was used in modified micelles to selectively deliver paclitaxel chemotherapy [125]. ImI modified micelles targeted α7 overexpressing tumour cells with higher specificity and efficacy. The selective targetting also resulted in low systemic toxicity and myelosuppression. This example paves the way for the application of selective conotoxins and analogues in targeted drug delivery for other diseases.

(5) Development as drug leads

α-Conotoxins have been considered for development as drug leads, however several challenges need to be overcome before the successful therapeutic application of these peptides [126]. The inherent selectivity profiles make α-conotoxins excellent tools for in vitro applications. However, selectivity windows of 10 to 30-fold may not always be sufficient for in vivo applications when considering the minimum effective doses required. Together with challenges associated with peptides such as in vivo bioavailability, stability etc., α-conotoxins perhaps better serve as structural scaffolds for rational drug design than as drug leads [4,24]. As starting structural scaffolds, α-conotoxins have resulted in lipophillic analogues with improved bioavailability, selenocysteine analogues with improved proteolytic resistance, and analogues with improved selectivity, such as ImI-dendrimers and TP-2212-59. The latter was developed using a combinatorial library with α-conotoxin BuIA as the starting template [124,127]. TP-2212-59 is reported to have over 1000-fold selectivity for α3β4 subtype over the α7, α3β2, and α4β2 subtypes [127]. Another emerging factor to be addressed in order to consider α-conotoxins as drug leads is, its pharmacological characterisation across a wider range of targets. Although α-conotoxins have been reliably demonstrated to be selective nAChRs inhibitors, the pharmacological profiles of some exceptions, such as Eu1.6, pl14a, µCnIIC, and α-Vc1.1 warrant a comprehensive screening of α-conotoxins against a wider range of targets. Vc1.1 was considered as a drug lead for its antinociceptive effects in animal models, however, its mechanism of action is highly debated with two proposed mechanisms of action via (1) the α9α10 v/s (2) GABA_B_ receptors [128,129,130].

## 6. Conclusions

α-Conotoxins are the largest group of natural product peptide inhibitors of the nAChRs. These disulfide-stabilised and highly structured molecules [2,126] have naturally engineered potency at nAChRs in the nM–pM range and target a broad range of nAChR subtypes, making them excellent tools to dissect the physiological and pathological functions of the diverse nAChR subtypes [24,42,43,131]. Their abundant expression across cone-snail species has provided a natural combinatorial library of peptides with variable primary sequences, structures, pharmacology, and mode of action at nAChRs [2,126,131,132]. This library has underpinned synthetic chemistry approaches that are focused on rational development of nAChR targeted lead molecules, research tools, and development of alternative/allosteric strategies for selective nAChR inhibition. Application of α-conotoxins have also extended towards the development of stable peptidomimetics for other receptor types, such as the glucagon-like peptide-1 receptor as well as a medium of targeted drug delivery in breast cancer [123,125]. High-throughput venomics approaches have accelerated identification of α-conotoxins inhibiting other receptor classes, which promise to provide first-in-class molecules targeting multiple receptor classes to dissect cross-talk in complex cellular mechanisms underlying conditions such as chronic pain [13]. 

## Figures and Tables

**Table 1:** Non-classical α-conotoxins and their pharmacology.

Superfamily	Sequence, Cysteine Framework and Connectivity	Pharmacology
**A superfamily**		
AuIB	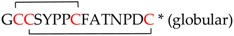	non-competitive inhibitor of the α3β4 [99]
AuIB	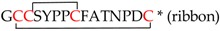	10-fold more potent in rat parasympathetic ganglions than the globular isomer
AusIA	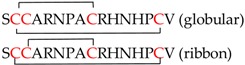	Defines a new 5/5 subclass. Both globular and ribbon isomers are equipotent at α7 [101]
ImII	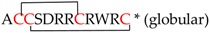	Lacks conserved proline in loop 1. Allosteric inhibitor of the α7
LtIA	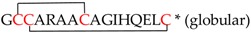	Ala-Xaa-Ala motif substitutes the conserved Ser-Xaa-Ser motif. Competitive blocker with a shallow binding pocket.
Lp1.1	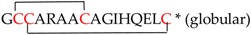	Ala-Xaa-Ala motif substitutes the conserved Ser-Xaa-Ser motif
MrIC	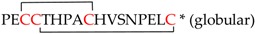	State dependent activator of the α7
Eu1.6	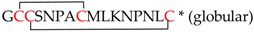	α-conotoxin inhibiting Cav 2.2 [107]
**S superfamily**		
RVIIIA	KCNFDKCKGTGVYNCG(Gla)SCSC(Gla)GLHSCRCTYNIGSMKSGCACICTYY	Atypical cysteine framework. Cysteine connectivity unknown. No C-terminal amidation. Two γ-carboxyglutamates. Broad selectivity: α7, α6/α3β2β3, α3β2 and to a smaller extent the α3β4 and α4β2 [9]
GVIIIA	GCTRTCGGOKCTGTCTCTNSSKCGCRYNVHPSG(BTr)GCGCACS *	Cysteine connectivity unknown. C-terminal amidation and bromo-tyrosine present as post-translational modification. Selective α9α10 inhibitor
**D superfamily**		
VxXXA	DVQDCQVSTOGSKWGRCCLNRVCGPMCCPASHCYCVYHRGRGHGCSC	Dimeric peptides. Allosteric inhibitors of the α7, α3β2 and α4β2.
VxXXB	DD(Gla)S(Gla)CIINTRDSPWGRCCRTRMCGSMCCPRNGCTCVYHWRRGHGCSCPG	
VxXXC	DLRQCTRNAPGSTWGRCCLNPMCGNFCCPRSGCTCAYNWRRGIYCSC	
GeXXA	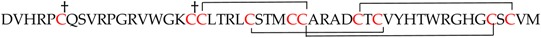	Dimeric peptide. Allosteric inhibitors of the α9α10. ‘Lid covering’ binding mode.
**B3 superfamily**		
VxXXIVA	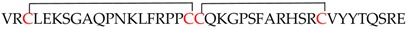	Potency dependent on disulfide connectivity—[1,2] 1.2 µM > [1,3] 3.9 µM > [1,4] > 30 µM, suggesting that the novel cysteine framework is important for its mode of action [26]
**O1 superfamily**		
GeXIVA	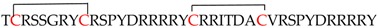	Inhibits α9α10 nAChR subtype with no activity at the Cav channels [29]. only four cysteine residues as opposed to 6 Potency: bead isomer > ribbon isomer > globular isomer Highly charged molecule. Allosteric mode of action.
**T superfamily**		
TxVC	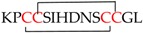	α4β2 inhibitor. Members of this superfamily typically target Ca_v_ channels.
**J superfamily**		
pl14a	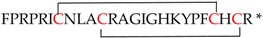	Targets Kv1.6 and α3β4
**M superfamily**		
CnIIIC	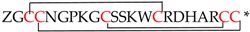	Targets Nav 1.2, 1.4 together with α3β2

(*) C-terminal amidation, (Gla) Gamma carboxylic glutamic acid, (BTr) bromotyrosine, (†) forms inter-chain disulfide bonds (Z) Pyroglutamic acid.
